# Comparative transcriptome analysis provides insight into regulation pathways and temporal and spatial expression characteristics of grapevine (*Vitis vinifera*) dormant buds in different nodes

**DOI:** 10.1186/s12870-020-02583-1

**Published:** 2020-08-26

**Authors:** Lingfei Shangguan, Mengxia Chen, Xiang Fang, Zhenqiang Xie, Peijie Gong, Yuxiang Huang, Zicheng Wang, Jinggui Fang

**Affiliations:** 1 Department of Horticulture, Nanjing Agricultural University, Nanjing, 210095 Jiangsu Province China; 2 Fruit Crop Genetic Improvement and Seedling Propagation Engineering Center of Jiangsu Province, Nanjing, 210095 China; 3 Department of Agriculture and Horticulture, Jiangsu Vocational College of Agriculture and Forestry, Jurong, 212499 Jiangsu Province China

**Keywords:** Grapevine, Bud dormancy, RNA-seq, Temporal and spatial expression

## Abstract

**Background:**

Bud dormancy is a strategic mechanism plants developed as an adaptation to unfavorable environments. The grapevine (*Vitis vinifera*) is one of the most ancient fruit vine species and vines are planted all over the world due to their great economic benefits. To better understand the molecular mechanisms underlying bud dormancy between adjacent months, the transcriptomes of ‘Rosario Bianco’ grape buds of 6 months and three nodes were analyzed using RNA-sequencing technology and pair-wise comparison. From November to April of the following year, pairwise comparisons were conducted between adjacent months.

**Results:**

A total of 11,647 differentially expressed genes (DEGs) were obtained from five comparisons. According to the results of cluster analysis of the DEG profiles and the climatic status of the sampling period, the 6 months were divided into three key processes (November to January, January to March, and March to April). Pair-wise comparisons of DEG profiles of adjacent months and three main dormancy processes showed that the whole grapevine bud dormancy period was mainly regulated by the antioxidant system, secondary metabolism, cell cycle and division, cell wall metabolism, and carbohydrates metabolism. Additionally, several DEGs, such as *VvGA2OX6* and *VvSS3*, showed temporally and spatially differential expression patterns, which normalized to a similar trend during or before April.

**Conclusion:**

Considering these results, the molecular mechanisms underlying bud dormancy in the grapevine can be hypothesized, which lays the foundation for further research.

## Background

Dormancy is an adaptive strategy of perennial woody plants to temporarily suspend visible growth to better endure harsh climatic conditions, which thus affects vegetative growth and fruit production in the following season [1]. Dormancy can mainly be divided into three stages depending on the transition phases: paradormancy, endodormancy, and ecodormancy [2]. Díaz-Riquelme et al. [3] used the same classification in research along the grape bud annual cycle. Initially, most research focused on the analysis of physiological and biochemical changes during bud dormancy. This kind of research includes changes in the form and content of water inside the buds, as well as the hormones present (such as ABA and GA) and carbohydrates (such as starch and sucrose) [4–9].

Recently, RNA-sequencing (RNA-seq) technology has been widely applied in pear [10, 11], grapevine [1, 12, 13], peach [14], Japanese apricot [15], sweet cherry [16, 17], kiwifruit [18], and litchi [19]. In addition, the wide application of proteomics technologies, such as isobaric tag for relative and absolute quantitation (iTRAQ) and tandem mass tags (TMT) [20–22], has achieved significant progress in dormancy regulative network research at the protein level. Dormancy-related regulatory pathways have been shown to be involved in the regulation of energy metabolism [21], phytohormones [1, 23, 24], cell division and growth [25], carbohydrate metabolism [12], signal transduction, oxidative stress [26, 27], secondary metabolism [22], and epigenetic control [14, 28]. A large number of coordinating key genes that regulate signaling pathways were identified. For example, *DORMANCY ASSOCIATED MADS-BOX* (*DAM*) and the flowering genes *FLOWERING TIME* (*FT*), *CENTRORADIALIS 1* (*CENL1*), and *SOC1* (*SUPPRESSOR OF OVEREXPRESSION OF CO1*) have been suggested to regulate endodormancy [29–33]. During both the induction and release of endodormancy, the *DAMs* expression profile indicated the possible existence of dose-dependent inhibitors of bud burst [14, 34].

As one of the most ancient fruit vine species, the fruits of the grapevine are mainly divided into table grapes, wine grapes, or both, and are cultured worldwide due to their great economic benefits. According to the results of a study on the ‘Fujimori’ grape, in southeast China, the winter dormancy period of grape wines could be divided into an initial dormancy period (early December), a deep dormancy period (early January), and a late dormancy period (early February) [35]. At the end of February, buds meet the chilling requirement and blossom in the following spring [1]. Much effort focused on illuminating the genetic network and elucidating the underlying molecular mechanism of bud dormancy of grapes and other fruit crops, in which thousands of genes are involved [12]. However, it still remains unclear how metabolic pathways change inside the grape buds between adjacent months from the onset of bud dormancy in early winter to the onset of bud germination in early spring. This might be due to the small number of samples or the extended sampling time span, both of which hinder the accurate description of changes of the metabolic pathways in the overwintering process of grape winter buds. Taking the studies of Min et al. [12] and Khalil-Ur-Rehman et al. [1] as examples, the sampling time intervals were either three or 6 months, which makes it difficult to describe the changing law of the specific regulation and the means to control grape buds during the short overwintering period. It is vital to comprehend the mechanisms of the onset and release of grape bud dormancy due to its significant impact on production. Therefore, to explore an intuitionistic dynamic metabolic change of grape buds from the dormancy induction stage through the deep dormancy period and to the dormancy release phase, three nodes of grape winter buds (top, center, and bottom) were collected every month from November to April of the following year. RNA-seq technology was employed to analyze the dynamic metabolic changes of dormant buds and the related gene expression trends (including novel genes) between adjacent months within six time points. At the same time, the temporal and spatial gene expression differences of different node buds in the same sampling time window were also preliminarily explored. These findings open a way for more in-depth and detailed studies of the dormancy mechanisms of grapes and other fruit trees, and also provide new insight into the study of spatial and temporal differential expression phenomena.

## Results

### Basic environmental conditions of sampling

**Fig. 1:**
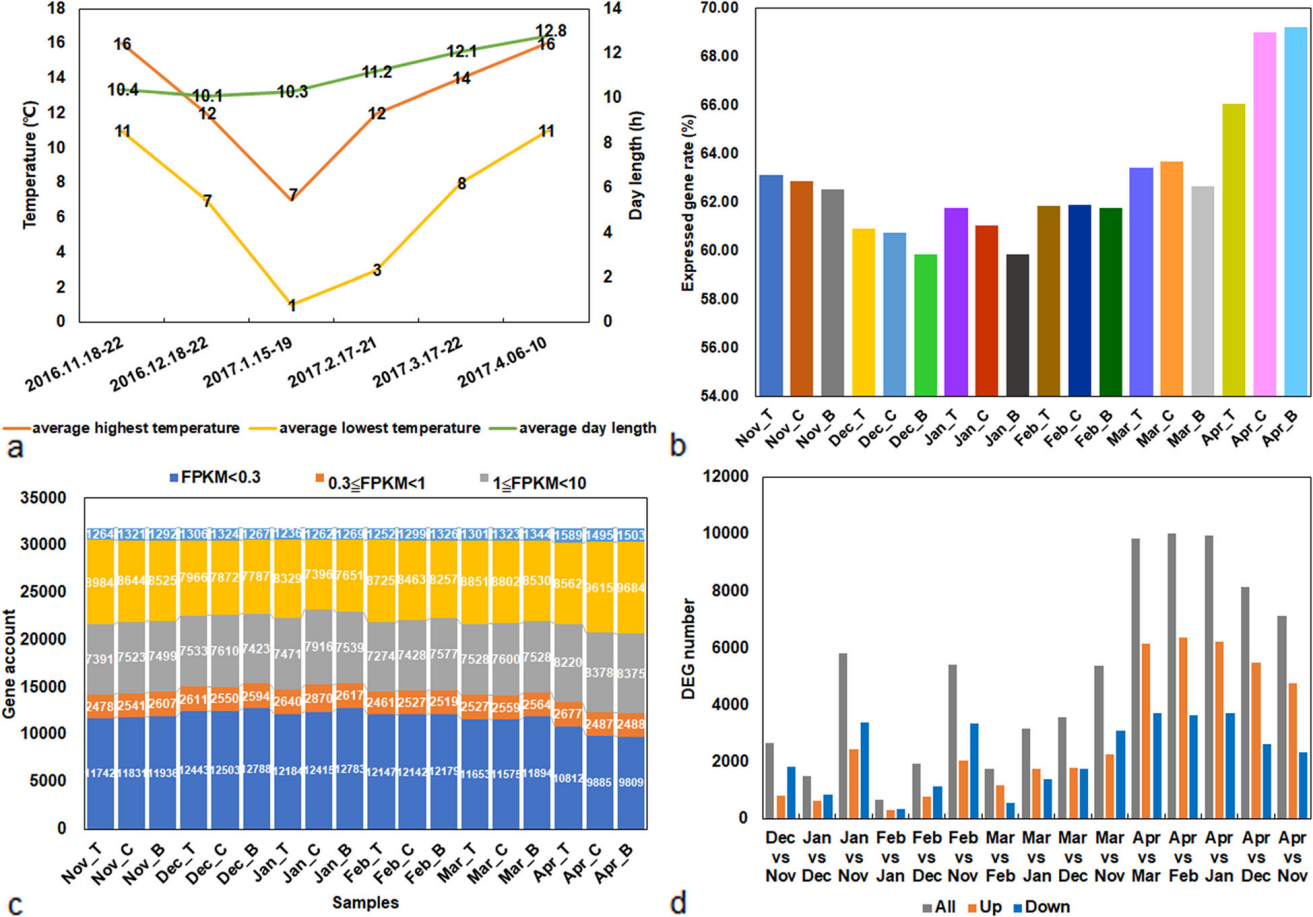
Weather conditions of sampling and gene expression information. **a** Average temperature and day length of sampling. **b** Rate of expressed genes based on all genes. **c** Gene numbers of different FPKM values. **d** DEG numbers of each pair-wise comparison

From November (Nov), 2016, to April (Apr), 2017, the shortest average day length was 10.1 h in December (Dec), followed by an extension of only 0.2 h in January (Jan), while the average day length increased to 12.8 h in Apr. The mean temperature showed a tendency to decrease first and then increase, and both the lowest values appeared in Jan, 2017, which were 7 °C (mean highest temperature) and 1 °C (mean lowest temperature), respectively (Fig. 1a, Table 1). Moreover, the humidity on the sampling day changed from 30 to 87% (Table 1).

**Table 1:** Weather conditions of the sampling

Date	Average highest temperature	Average lowest temperature	Humidity for the day of sampling	Average day length
2016.11.18–22	16 °C	11 °C	87%	10:24:31
2016.12.18–22	12 °C	7 °C	64%	10:03:11
2017.1.15–19	7 °C	1 °C	49%	10:19:32
2017.2.17–21	12 °C	3 °C	30%	11:11:30
2017.3.17–22	14 °C	8 °C	70%	12:05:53
2017.4.06–10	16 °C	11 °C	63%	12:45:02

### Global analysis for RNA-Seq data

RNA-seq data was generated from various dormancy stages and different nodes of grape buds. A total of 36 cDNA libraries were constructed from grape buds, generating 1595.9 million raw sequencing reads. After a series of strict quality controls and data trimming, 1593.9 million high-quality clean reads, containing 268.83 G nucleotide sequences, were obtained in total with rates of more than 99.78%. More than 88% of high-quality reads from individual sample types were mapped to the grapevine reference genome, and 86.01% or more were mapped uniquely (Supplementary Table 1).

Only 59.86 to 69.21% genes (31,858 genes in total) showed a FPKM value ≥0.3 (Fig. 1b and c, Supplementary Tables 2 and 3). More than half of the genes had transcriptional values exceeding 1 (Fig. 1c, Supplementary Tables 2 and 3). These results indicated a good representation of the grape dormant bud RNA-seq data.

Based on the square of the Pearson correlation coefficient, the degree of sample repeatability was evaluated, which was represented by the R^2^ value. Overall, all biological repetitions showed highly correlated expressions, and further statistical analyses could be performed based on this dataset. Additionally, the R^2^ values of Dec vs Nov, Jan vs Dec, February (Feb) vs Jan, and March (Mar) vs Feb were high, while the R^2^ value of Apr vs Mar was relatively low (Supplementary Fig. 1). These results indicated a smooth transition during grape bud dormancy release, and a dramatic change during the stage between Mar and Apr which had a more complex transcriptome.

### Pair-wise comparisons and clustering analysis of DEG profiles

In this study, a total of 11,647 DEGs were identified, which were subjected to a pair-wise comparison between different stages to investigate global changes in gene expression during bud dormancy transition. The minimum number of DEGs (665) was found in Feb vs Jan with 321 up- and 344 down-regulated DEGs. In contrast, the highest number (10,332 DEGs) was found in the comparison of Apr vs Feb with 6373 up- and 3659 down-regulated DEGs, followed by Apr vs Jan, with 9957 DEGs in total, 6225 of which were up- and 3732 were down-regulated. Additionally, significant differences were found in the comparisons of Apr with all other 4 months (Fig. 1d, Table 2).

**Table 2:** Numbers of DEGs in each comparison

Comparison group	DEG number		
	All	Up	Down
Dec vs Nov	2655	832	1823
Jan vs Dec	1502	654	848
Jan vs Nov	5839	2452	3387
Feb vs Jan	665	321	344
Feb vs Dec	1937	791	1146
Feb vs Nov	5441	2066	3375
Mar vs Feb	1754	1196	558
Mar vs Jan	3191	1781	1410
Mar vs Dec	3571	1803	1768
Mar vs Nov	5376	2277	3099
Apr vs Mar	9849	6134	3715
Apr vs Feb	10,032	6373	3659
Apr vs Jan	9957	6225	3732
Apr vs Dec	8137	5483	2654
Apr vs Nov	7136	4778	2358

Note: RNA-seq, |log2FC| ≥ 1, padj< 0.05

**Fig. 2:**
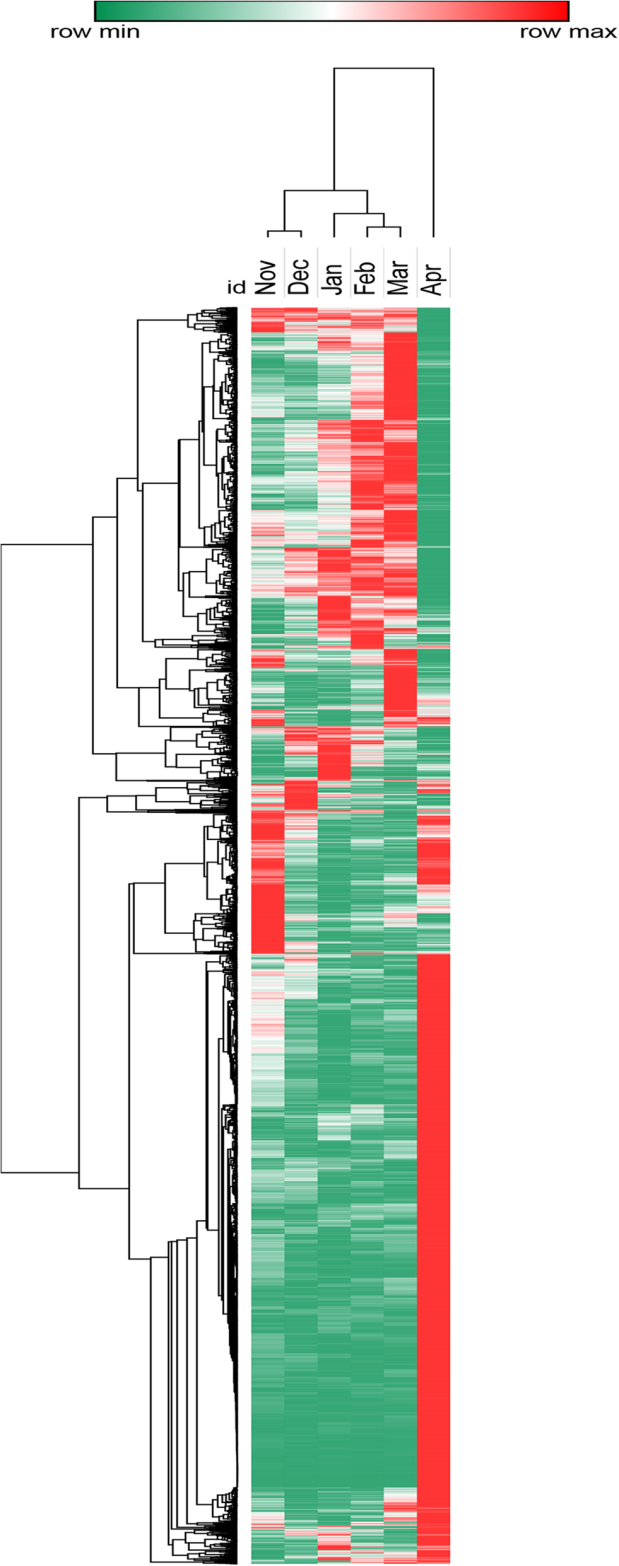
Clustering analysis of total DEGs during grape dormancy. The cluster display expression patterns for a subset of DEGs in 6 months. Each column represents an experimental condition and each row represents a gene. Red means the highest expression of a DEG in 6 months and green means the lowest

Cluster analysis of all 11,647 DEGs showed that the samples collected per month clustered into three main groups according to their expression changing patterns (Fig. 2). These include samples collected in Nov and Dec, in Jan to Mar, and in Apr. The clustering results indicated that Jan and Mar might be turning points in the process of dormancy transition and Apr might be the initiation of grapevine bud dormancy release. Therefore, based on the results of cluster analysis and the climate of the sampling period, the 6 months were roughly divided into three main stages, i.e., Nov to Jan, Jan to Mar, and Mar to Apr. The specific comparative contents are detailed in the following.

### Functional annotations of total DEGs during grape dormancy

**Fig. 3:**
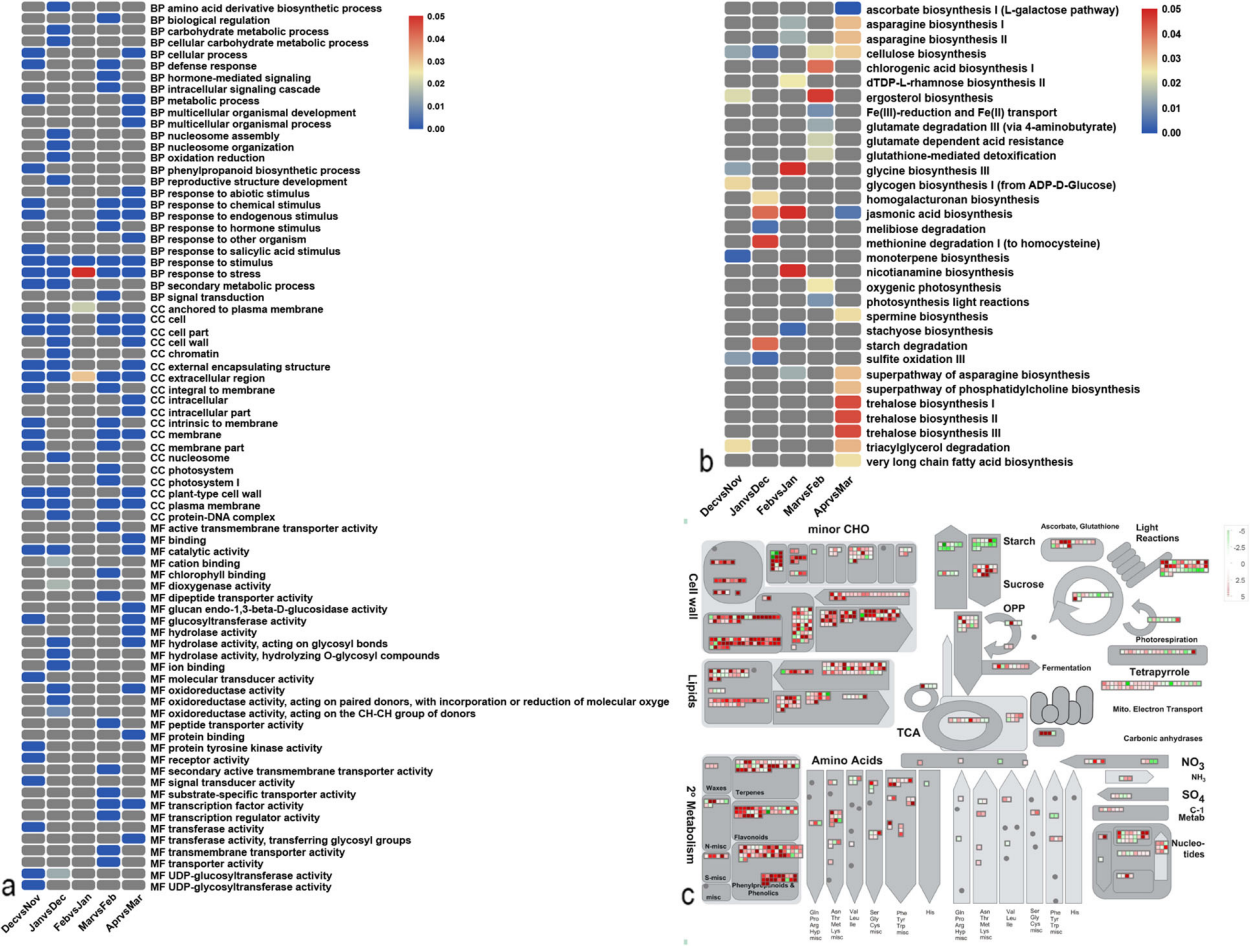
Functional annotations of total DEGs during grape dormancy. **a** The top 10 significantly enriched GO terms of each pair. The legend indicates 0 ≤ FDR ≤ 0.05. **b** The significantly enriched KEGG terms of each pair. The legend indicates 0 ≤ *p*-value ≤0.05. **c** Metabolism overview of Apr vs Mar. Red indicates up-regulation and green indicates down-regulation

GO, KEGG, and MapMan analyses were conducted for the DEGs. The significantly enriched GO terms were involved in biological process (BP), cellular component (CC), and molecular function (MF). Figure 3a shows the top 10 significantly enriched terms of each GO category in month-to-month comparisons. The results of GO analysis indicated that the terms “response to stimulus”, “response to stress”, “response to chemical stimulus”, and “response to endogenous stimulus” were predominant categories of BP, which were significantly enriched in more than three pair-wise comparisons. In CC terms, “extracellular region”, “cell”, “cell part”, and “plasma membrane” were enriched in four or more comparisons. Finally, in MF terms, only “catalytic activity” was enriched in three pairs, while “hydrolase activity, acting on glycosyl bonds”, “oxidoreductase activity”, and “transcription factor activity” were identified in two comparisons (Fig. 3a; Supplementary Table 4).

KEGG analysis showed that “cellulose biosynthesis”, “jasmonic acid biosynthesis”, and “asparagine biosynthesis II and III” were significantly enriched in more than two compared combinations (Fig. 3b, Supplementary Table 5). Moreover, several important pathways stood out in one of the comparisons. Examples are “ascorbate biosynthesis I (L-galactose pathway)” in Apr vs Mar, “photosynthesis light reactions” and “oxygenic photosynthesis” in Mar vs Feb, as well as “starch degradation” in Jan vs Dec. Several pathways did not meet the condition of significant enrichment (*p*-value ≤0.05), but also played an indispensable role during the dormancy process. Examples are “ethylene biosynthesis from methionine” (minimum p-value 0.052409 in Apr vs Mar), “S-adenosylmethionine biosynthesis” (minimum p-value 0.116349 in Feb vs Jan), and “starch biosynthesis” (minimum p-value 0.0931243 in Dec vs Nov) (Supplementary Table 5).

MapMan analysis was also conducted to provide an overview of changes in a number of important metabolic pathways and relevant functional groups during the dormancy transition process. The DEGs were found to be involved in many functions, and the top five categories were “cell wall”, “starch-sugar metabolism”, “light reactions”, “lipids”, and “secondary metabolism” (Fig. 3c, Supplementary Fig. 2A-D). For instance, DEGs involved in the categories “cell wall”, “lipids”, “secondary metabolism”, and “light reactions” were mainly down regulated, while the category “starch-sugar metabolism” was mainly up-regulated in Dec vs Nov (Supplementary Fig. 2D). Apr vs Mar, predictably, had the highest number of DEGs, annotated to the items of Mapman. Except for the obvious down-regulation of starch metabolism, the other above-mentioned pathways were strongly up-regulated (Fig. 3c).

### Differentially expressed genes (DEGs) involved in the antioxidant system during bud dormancy

**Fig. 4:**
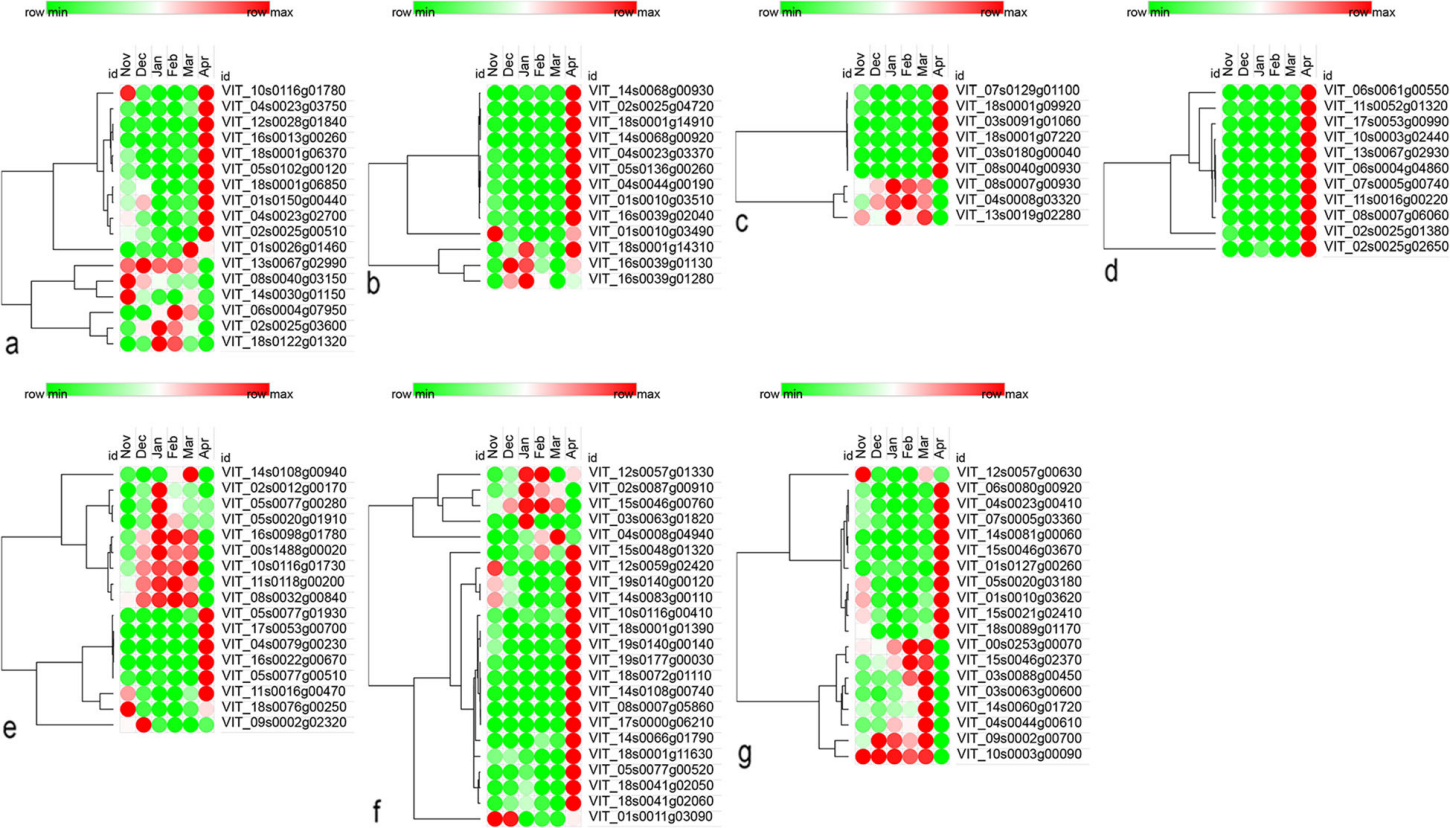
Expression analysis of key DEGs involved in different pathways. Each column represents an experimental condition and each row represents a gene. Red means the highest expression of a DEG in 6 months and green means the lowest. **a** Key DEGs in the antioxidant system. **b** Key DEGs in secondary metabolism. **c** Key DEGs in cell cycle and cell division. **d** Key DEGs in cell wall metabolism. **e** Key DEGs in the starch-sucrose metabolism. **f** Key DEGs in phytohormone pathways. **g** Key DEGs in other pathways

Oxidative stress has been suggested to be an important factor in promoting the release of grapevine bud dormancy [36], including the generation and scavenging of ROS during the bud dormancy transition. In this study, a total of 136 DEGs were involved in intracellular redox equilibrium, 98 of which were up-regulated in Apr compared with the dormancy period, while 38 DEGs were down-regulated (Supplementary Table 6). DEGs of *peroxiredoxin* (*VvPOD*; e.g., VIT_10s0116g01780, VIT_18s0001g06850, and VIT_12s0028g01840), *ascorbate peroxidase* (*VvAPX*; e.g., VIT_04s0023g03750 and VIT_18s0001g06370), *glutathione peroxidase* (*VvGPX2*; VIT_05s0102g00120), and *thioredoxin* (*VvTrx*; e.g., VIT_01s0026g01460 and VIT_04s0023g02700) all maintained low expression levels before dormancy release and increased expression levels in Apr (Fig. 4a, Supplementary Table 6). In contrast, *VvAPX2* (VIT_08s0040g03150), *VvGPX6* (VIT_02s0025g03600), *manganese superoxide dismutase 1* (*VvMSD1*; VIT_13s0067g02990 and VIT_06s0004g07950), *copper chaperone for SOD1* (*VvCCS*; VIT_14s0030g01150), and *catalase* (*VvCAT2*; VIT_18s0122g01320) were significantly suppressed in Apr. Among these, *VvCAT2* increased from Nov and peaked in Jan, then gradually decreased until Apr. Moreover, 21 out of 36 glutathione-S-transferase (GST) encoding genes were down-regulated in Apr, but highly expressed during dormancy. However, two *Respiratory burst oxidase homolog* (*Rboh*; VIT_02s0025g00510 and VIT_01s0150g00440) genes, which encode key proteins for the synthesis of ROS [37], showed higher expressions (Fig. 4a, Supplementary Table 6).

Furthermore, a gene (VIT_08s0007g00330) encoding metallothionein (MT) was found and showed high expression levels over 6 months, peaked in Mar, and then significantly decreased in Apr. This was related to the detoxification of heavy metals, the homeostasis of essential metal ions [38], and gene regulation during chilling accumulation in grape [25], peach [39], and *poplar* buds [40] and might play a role in antioxidant processes (Fig. 4a, Supplementary Table 6).

### DEGs involved in secondary metabolism during bud dormancy

The secondary metabolism has been reported to be involved in the bud dormancy process of the grapevine [12]. A total of 218 DEGs were involved in flavonoids, phenylpropanoids, isoprenoids, alkaloid-like, simple phenols, and wax metabolism, 57% of which had their highest expression in Apr, while 15% had their lowest expression in Apr (Supplementary Table 6).

Thirty-four out of 50 DEGs relevant to the flavonoid metabolism showed higher abundance in Apr compared with the dormancy period (Supplementary Table 6). DEGs encoding anthocyanidin synthase (*VvANS*; VIT_02s0025g04720), putative flavanone 3-hydroxylase (*VvF3H*; VIT_18s0001g14310 and VIT_04s0023g03370), and chalcone synthase (*VvCHS*; VIT_14s0068g00930, VIT_14s0068g00920, and VIT_05s0136g00260) were highly induced in Apr. In particular, *VvANS* showed nearly 40-fold increase in Apr and its lowest expression level was recorded in Dec. Forty-six out of 66 DEGs involved in phenylpropanoids metabolism showed varying degrees of up-regulation in Apr. Examples are *CINNAMYL ALCOHOL DEHYDROGENASE 9* (*VvCAD9*; VIT_18s0001g14910 and VIT_04s0044g00190), *4-coumarate-CoA ligase* (*Vv4CL*;VIT_16s0039g02040), *caffeoyl-CoA O-methyltransferase* (*VvCCoAMT*; VIT_01s0010g03510 and VIT_01s0010g03490), and *phenyl alanine ammonia-lyase* (*VvPAL*; VIT_16s0039g01130 and VIT_16s0039g01280) (Fig. 4b, Supplementary Table 6).

### DEGs involved in cell cycle and cell division during bud dormancy

During bud dormancy, most cells are repressed at the G1 phase in the cell cycle [41, 42]. Thus, once buds resume their growth in spring, the genes that control the transition of the G1-to-S phase, such as *D-type cyclins* (*CYCDs*), may be strongly activated before budbreak [42, 43]. In this study, 77 DEGs involved in cell cycle and cell division were screened during dormancy transition, 58 of which were highly induced in Apr, while 19 were down-regulated in Apr. DEGs were involved in cell cycle regulation, such as *VvCYCDs* (VIT_07s0129g01100, VIT_03s0091g01060, VIT_18s0001g09920, VIT_18s0001g07220, and VIT_03s0180g00040) and *VvCYCB1;2* (VIT_08s0040g00930). These maintained low expression levels during the dormancy period, which sharply increased when dormancy began to release in Apr. In addition, almost 60% of the DEGs associated with cell division had significantly increased expression in Apr, although the expression magnitudes were low throughout the whole process of dormancy. However, the remaining genes that were down-regulated in Apr had relatively higher expression levels during the dormancy period. For instance, genes (VIT_08s0007g00930, VIT_04s0008g03320, and VIT_13s0019g02280) that encode the regulator of chromosome condensation (RCC1) family protein were markedly repressed in Apr (Fig. 4c, Supplementary Table 6).

### DEGs involved in cell wall metabolism during dormancy transition

In total, 284 DEGs associated with cell wall metabolism were identified. Nearly 82% of these showed down-regulation during the dormancy period, and up-regulated during the dormancy release in Apr, while only about 18% showed the opposite trend (Supplementary Table 6). The metabolic processes of the cell walls that were activated during the dormancy release phase mainly included cellulose synthesis, synthesis of various cell wall degradation related enzymes (e.g., cellulases, beta-glucanases, and pectate lyases), synthesis of cell wall modification related enzymes and proteins (e.g., xyloglucan endotransglucosylase/hydrolase and expansin), and cell wall precursor synthesis. For instance, the expression levels of xyloglucan endotransglucosylase/hydrolase (*VvXTHs*; e.g., VIT_10s0003g02440, VIT_06s0061g00550, and VIT_11s0052g01320), expansin encoding genes (e.g., VIT_13s0067g02930, VIT_06s0004g04860, and VIT_17s0053g00990), and endoglucanase (endo-1,3(4)-beta glucanase) encoding genes (e.g., VIT_07s0005g00740, VIT_02s0025g01380, VIT_02s0025g02650, VIT_08s0007g06060, and VIT_11s0016g00220) at the beginning of the dormancy release and sprout phase in Apr were hundreds of times higher than during the dormancy period (Fig. 4d, Supplementary Table 6).

### DEGs associated with the starch-sucrose metabolism during bud dormancy

Carbohydrates, such as starch and soluble sugars, showed varying compositions during bud dormancy transition in various species including grape [12, 44] and pear [45]. In the present study, 56 DEGs were related to starch and sucrose, including those involved in carbohydrate synthesis, metabolism, transportation, and signaling. On the one hand, nine starch synthesis DEGs and 18 starch metabolic DEGs were found to maintain high expression levels before Apr, which dramatically decreased in Apr (Supplementary Table 6). For example, the four paralogs of *STARCH SYNTHASE* (*VvSS*; VIT_16s0098g01780, VIT_00s1488g00020, VIT_14s0108g00940, and VIT_10s0116g01730) maintained relatively high expression levels between Nov and Mar, peaked either in Jan or Mar, and then sharply decreased in Apr (Fig. 4e, Supplementary Table 6). The FPKM values of starch metabolic genes, e.g., *BETA-AMYLASE* (*VvBAM*; VIT_05s0077g00280, VIT_02s0012g00170, and VIT_05s0020g01910), increased from Nov to Jan and then decreased, ranging from 1300 to 2800 in Jan (Fig. 4e, Supplementary Table 6).

On the other hand, sucrose metabolic genes, e.g., four *SUCROSE SYNTHASE* (*VvSUS*; VIT_05s0077g01930, VIT_11s0016g00470, VIT_17s0053g00700, and VIT_04s0079g00230) and three invertases (VIT_05s0077g00510, VIT_16s0022g00670, and VIT_09s0002g02320), showed significantly increased expression in Apr compared with the dormancy period. In addition, the expression of sucrose-proton symporter 2 (*VvSUC2*; VIT_18s0076g00250), gradually decreased from Nov to Feb and then increased until Apr, while sucrose synthetic genes, including sucrose phosphate synthase (*VvSPS1*; VIT_11s0118g00200) and sucrose phosphate synthase (*VvSPP*; VIT_08s0032g00840) showed the opposite changing pattern of *VvSUC2* (Fig. 4e, Supplementary Table 6)*.*

### DEGs related to phytohormone-related pathways during dormancy transition

In total, 299 DEGs were identified to be involved in hormone synthesis, degradation, signaling, and transduction. Among these, 35 were related to abscisic acid (ABA) metabolism, 32 were related to gibberellin (GA) metabolism, 72 were related to auxin metabolism, 12 were related to cytokinin (CTK) metabolism, 92 were ethylene (ETH) related genes, 23 were jasmonate acid (JA) associated genes, 13 were salicylic acid (SA) associated genes, and 20 were brassinosteroid (BR) associated genes (Supplementary Table 6).

For example, the expression pattern of 9-cis-epoxy-carotenoid dioxygenase (*VvNCED4*; VIT_02s0087g00910), a key biosynthesis gene of ABA, first increased from Nov to Jan, peaked in Jan, and then gradually decreased to the lowest level in Apr (Fig. 4f, Supplementary Table 6). *VvXERICO* (VIT_12s0057g01330) was increasingly expressed from Nov to Feb, sharply decreased in Mar, and then almost tripled in Apr, which still remained lower compared with Feb and Jan (Fig. 4f, Supplementary Table 6). ABA inactivation related genes, such as *VvCYP707A4* (VIT_12s0059g02420) gradually declined from Nov to Jan, and then increased to the highest level in Apr, exhibiting the opposite pattern of *VvNCED4* (Fig. 4f, Supplementary Table 6).

GA biosynthetic genes, such as two *VvGA20ox* (VIT_18s0001g01390 and VIT_15s0048g01320) and five *VvGA2ox* (VIT_05s0077g00520, VIT_19s0177g00030, VIT_19s0140g00120, VIT_19s0140g00140, and VIT_10s0116g00410), had low expression levels during the dormancy period, and showed significant up-regulation in Apr. However, two *VvGA3ox* genes (VIT_15s0046g00760 and VIT_04s0008g04940) showed the opposite results than the genes above (Fig. 4f, Supplementary Table 6). In this study, five putative GA-responsive *GAST1 PROTEIN HOMOLOG* genes (*VvGASA*; VIT_18s0072g01110, VIT_14s0108g00740, VIT_14s0066g01790, VIT_08s0007g05860, and VIT_17s0000g06210) maintained extremely low expression levels between Nov and Mar, which then strongly increased in Apr (Fig. 4f, Supplementary Table 6).

JA is also a universal phytohormone that plays a key role in stress defense as well as seed germination, plant growth, abscission, and senescence [46]. Recently, attention has been focused on the involvement and regulation of JA in bud dormancy [47, 48]. JA biosynthesis associated genes exhibited a well-marked difference during dormancy development. *12-oxophytodienoate reductase 2* (*VvOPR2*; e.g., VIT_18s0041g02060 and VIT_18s0041g02050), *ALLENE OXIDE CYCLASE 4* (*VvAOC4*; VIT_14s0083g00110 and VIT_01s0011g03090), and *ALLENE OXIDE SYNTHASE* (*VvAOS*; VIT_18s0001g11630 and VIT_03s0063g01820) were all up-regulated in Apr, while they retained low expression levels during the dormancy period, especially between Jan and Mar (Fig. 4f, Supplementary Table 6).

### Transcription factor encoding genes

The bud dormancy status is positively or negatively regulated by transcription factors (TFs) that control various key gene expressions [49]. A total of 493 DEGs, which belong to 25 major TF families, were found to be involved in the dormancy process of grape buds (Table 3, Supplementary Table 7). For example, 80% WRKY TFs encoding genes showed high expression levels when bud dormancy started to release in Apr. Examples are *VvWRKY40* (VIT_09s0018g00240) and *VvWRKY41* (VIT_15s0046g01140), the expressions of which were 12 and 27 times higher in Apr than in the months with the lowest levels (Supplementary Table 7). 90% of bHLH family members showed relatively higher expression levels in Apr than between Dec and Feb, nearly 51% of which showed minimum expression in Jan (Supplementary Table 7). Almost 75% of MYB TFs were activated in Apr, although the expression magnitudes generally remained low through the whole process of dormancy development. For instance, *VvMYB15* and *VvMYB111* were found to be highly induced in Apr, while *VvMYB5* and *VvMYB61* were dramatically down-regulated (Supplementary Table 7). The AP2-like ABA repressor 1 (*ABR1*) gene, which is strongly responsive to ABA and functions as a negative regulator of ABA, was highly expressed in Nov, Dec, and Apr, while its expression was significantly lower between Jan and Mar (Supplementary Table 7).

**Table 3:** Numbers of transcription factors

TFs	Apr *vs* Mar		Mar *vs* Feb		Feb *vs* Jan		Jan *vs* Dec		Dec *vs* Nov		Total
	up	down	up	down	up	down	up	down	up	down	
MYB	58	12	7	3	3	3	5	13	3	27	87
bHLH	50	6	11	0	2	0	4	8	1	14	63
AP2	32	7	7	1	2	1	3	7	4	5	46
HB	17	19	11	1	1	0	2	3	3	9	42
WRKY	25	6	2	1	0	1	1	2	2	6	35
C2H2	18	13	6	0	0	2	2	2	3	3	33
bZIP	10	6	2	0	1	1	1	0	3	4	20
MYB-related	5	13	1	2	1	0	2	1	4	2	19
G2-like	5	7	5	2	0	1	3	0	1	3	16
C2C2-CO-like	2	11	1	0	1	1	4	0	4	0	15
GRAS	11	2	5	3	0	0	0	1	3	2	15
MADS	5	5	4	0	0	0	0	0	1	2	14
Dof	3	4	5	0	0	0	1	0	3	3	14
HSF	4	5	1	5	0	1	0	1	1	0	12
ARR-B	5	3	1	0	0	0	0	2	0	2	9
C2C2-GATA	9	1	0	0	1	2	1	2	0	2	9
Trihelix	2	3	1	0	1	0	1	1	1	2	9
C3H	3	5	1	0	0	0	0	0	0	0	8
ARF	2	4	2	0	0	1	1	0	0	1	7
TCP	4	1	0	0	2	1	0	0	1	1	7
NIN-like	1	3	0	0	0	0	0	0	0	1	4
B3	4	0	0	0	0	0	0	0	0	0	4
CPP	3	0	0	0	0	0	0	0	0	2	3
GeBP	0	1	0	0	0	0	0	0	0	0	1
NAC	0	1	0	0	0	0	0	0	0	0	1

Total = unredundant sum of the DEGs numbers in each group

The expression patterns of *DAMs* coincided with the transition of the endodormancy phase in peach and pear [50, 51]. *VvDAM2* (VIT_18s0001g07460) was identified in this study, which was not a DEG, but the overall expression pattern matched the assumption. The overall expression of *VvDAM2* (VIT_18s0001g07460) followed an increasing trend from Nov to Mar. It showed a slight decrease in Feb and the highest expression in Mar, which then decreased to the lowest level in Apr when bud dormancy began to release (Supplementary Table 7).

### DEGs involved in other dormancy-related metabolic pathways

A total of 90 DEGs were found to be related to photosynthesis, including those involved in photosystem I (PSI), photosystem II (PSII), and photosynthetic electron transport. Fifty-six DEGs maintained a relatively lower expression level during the dormancy period, and were then strongly up-regulated when buds began to break in Apr. Examples are PSI and PSII polypeptide subunits (e.g., VIT_14s0081g00060, VIT_06s0080g00920, VIT_05s0020g03180, and VIT_04s0023g00410) and the light harvesting complex of PSII (chlorophyll binding; e.g., VIT_12s0057g00630, VIT_01s0010g03620, and VIT_18s0089g01170) (Fig. 4g, Supplementary Table 6). However, genes involved in respiration were also found to be significantly differently expressed in this study. The expressions of a total of 17 of 25 DEGs of the TCA cycle were activated in Apr, comprising ATP citrate synthase (VIT_01s0127g00260) and malate dehydrogenase (VIT_07s0005g03360, VIT_15s0046g03670, and VIT_15s0021g02410) (Fig. 4g, Supplementary Table 6).

Autophagy is an evolutionarily conserved catabolic process [52]. Eight autophagy-related genes were found in this study. The expression levels of *VvATG101* (VIT_00s0253g00070), *VvATI* (VIT_15s0046g02370), *VvTOR* (VIT_03s0088g00450), *VvATG2* (VIT_03s0063g00600), and *VvATG13a* (VIT_04s0044g00610) showed higher expression during natural dormancy than during dormancy release in Apr. *VvATI* peaked in Feb (Fig. 4g, Supplementary Table 6).

In addition, three genes (VIT_09s0002g00700, VIT_14s0060g01720, and VIT_10s0003g00090) were similar to dormancy/auxin associated family proteins in *Arabidopsis*. These genes presented extremely high expression levels between Nov and Mar, which dramatically decreased when bud dormancy began to release in Apr (Fig. 4g, Supplementary Table 6).

### Analysis of novel genes

A total of 1249 novel genes were obtained from the sequencing result, and their functional prediction and GO annotations were generated via Blast2GO. Six hundred sixty-two of 1249 novel genes were DEGs, which were also annotated to BP, MF, and CC, such as “regulation of jasmonic acid mediated signaling pathway”, “response to gibberellin”, “response to abscisic acid”, “regulation of defense response”, “response to oxidative stress”, “carbohydrate metabolic process”, “signal transduction”, “oxidoreductase activity”, and “cell wall” (Supplementary Table 8). Specifically, these novel genes encode jasmonate-zim-domain protein 8 (Novel00062), peroxidase superfamily protein (Novel00457), late embryogenesis abundant protein (LEA; Novel00626), osmotin (Novel00636), *VvF3H* (Novel00736), *ALPHA-GLUCAN PHOSPHORYLASE 2* (*VvPHS2*; Novel01068), and cold shock domain protein 3 (Novel00454) (Supplementary Table 8).

Furthermore, several remarkable novel genes were identified, such as *autophagy 3* (*APG3*; Novel00335), which was not a DEG, but might play an important role in the process of bud dormancy (Supplementary Table 8). With the continuous improvement of sequencing methods and the genome quality, more so-called novel genes will be annotated in the grape genome.

### Validation of RNA-seq by RT-qPCR

**Fig. 5:**
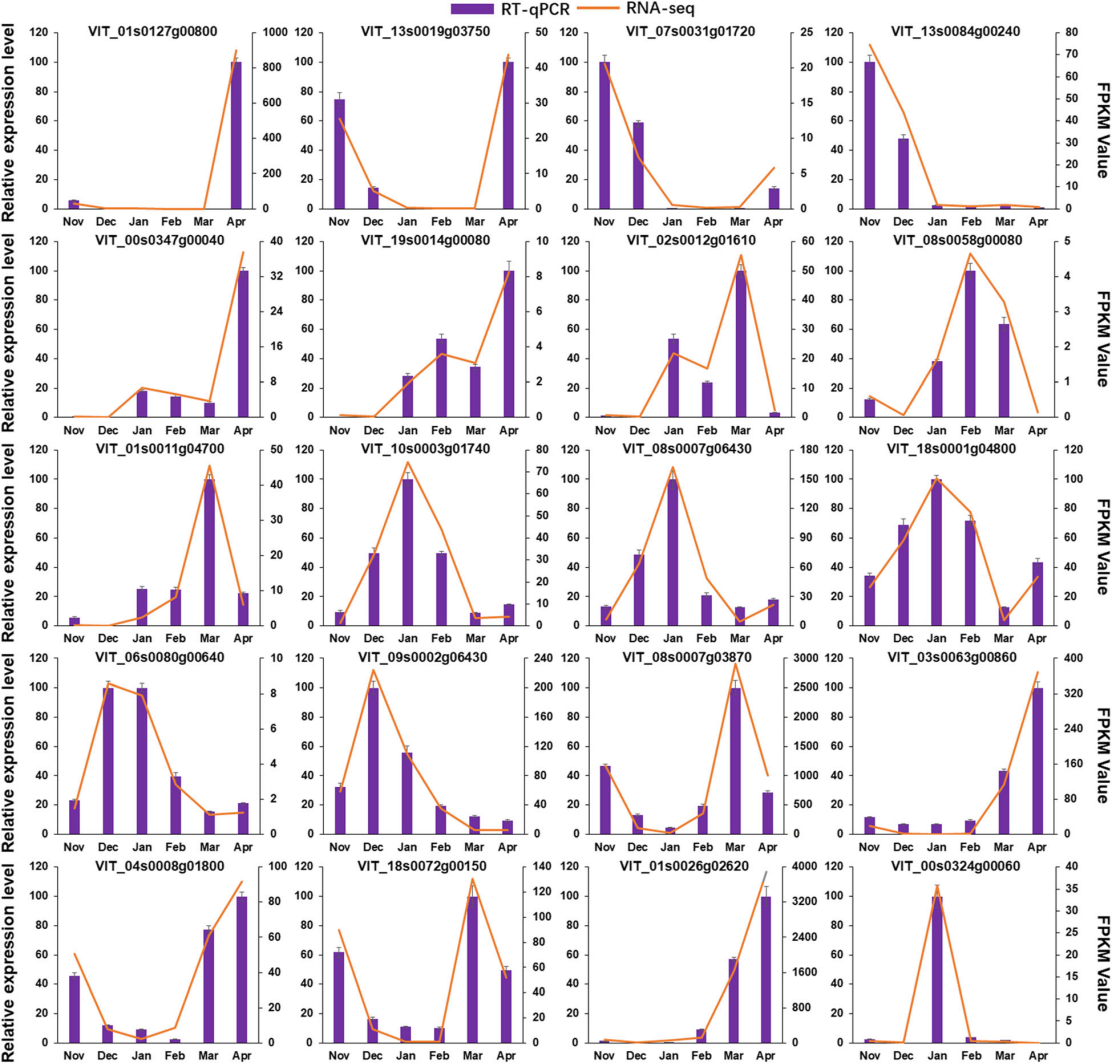
Validation of RNA-seq by RT-qPCR. The column chart and the main longitudinal coordinate represent the relative expression of RT-qPCR, while the broken line diagram and the secondary longitudinal coordinate represent the FPKM value of RNA-seq

To verify the precision and repetitiveness of the transcriptome analysis results, 20 DEGs were randomly selected for RT-qPCR analysis. Gene specific RT-qPCR primer pairs are listed in Table 4. Although few genes differed slightly in several months (e.g., VIT_01s0011g04700, VIT_06s0080g00640, and VIT_04s0008g01800), the 20 selected DEGs generally showed consistent expression patterns between RT-qPCR and RNA-Seq, indicating the reliability of expression data generated from RNA-Seq (Fig. 5).

**Table 4:** Primer pairs of RT-qPCR

Locus ID	Primer-F (5′-3′)	Primer-R (5′-3′)
VIT_01s0127g00800	GTTATTGTTGTTGGAGCCGGCA	CCCAGTTTGCTCCCATTTCCAC
VIT_13s0019g03750	AACTTCCCGAGTCCAAGAGAGC	GCATTGTATGAGAAGGAGCGCC
VIT_07s0031g01720	AAGTCATAGTCCTCGGCGACAG	CCTGCTGTGTCCCAGAGTTGTA
VIT_13s0084g00240	AACTTCCCTGACCTCTCCAAGC	AAGTGCCAGGAAGACGCAAAAG
VIT_00s0347g00040	GGGTGTGATCACGTTTGAGAGC	AGCCACCTTCTGGAACTTGTCA
VIT_19s0014g00080	AGGTTTTTGCTGCTCACTTCGG	CCATTAGCGGCTCCGGAAAATC
VIT_02s0012g01610	CCACGGCAGTTCTTGGAACTTC	TCCAATGGGACGTTTACTGGGT
VIT_08s0058g00080	AAGGCTGTTCTGTGAGAGGTCC	GCTGTCCACAAGCATCCTTCAG
VIT_01s0011g04700	CAGCACAAGGAAGAGCAAACGT	CACTGACTGTTACCCGGCTTTG
VIT_10s0003g01740	AAGCTGCTGGAAGAACAATGGC	AAGGGTAGCGAGCAGTGTCATT
VIT_08s0007g06430	CAGAGCTGGCATGGAGAAAACC	CTGCATTGTGTTCCTGAGCCAT
VIT_18s0001g04800	AGAAGGCACAGTCAACAGGGAA	TCGCTGAACATGGTGGTGTACT
VIT_06s0080g00640	GCTTTCACTCGAGGCAACCAAA	TCTAACATTTTGCCCGCCCTTG
VIT_09s0002g06430	CATGGTCGAGATCTGCAACTGC	GAGAGGAGAGTGTAGGCAGTGG
VIT_08s0007g03870	TATACGAGCCATTTGCACCACC	AGTCATCCACTCACCCATCACC
VIT_03s0063g00860	GGCAGCTGAGGATCCATCATCT	CCACTTTGTTCCAGGCATACGG
VIT_04s0008g01800	CTCCCCAAGAGAGCTGGTTTGA	ATTTCATTGTCTGTTCGCCCCG
VIT_18s0072g00150	AGGAGCAGCTTCAAGTGGATCA	AGAGCCACACACCATCATCCAA
VIT_01s0026g02620	AAACACAGCAGCTTTGAGCACA	GAGGGCATTGTTTGGTGGACAG
VIT_00s0324g00060	AAAGCGCCATGACATTGAAGCA	GCAGAACCTCCTTGACGAGTCT

### Temporal and spatial expression of DEGs in different nodes

**Fig. 6:**
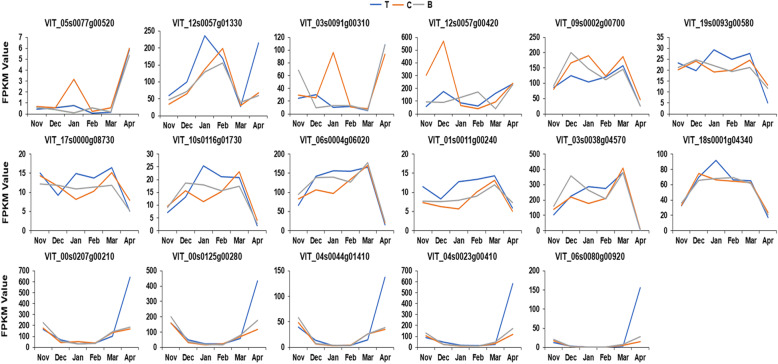
Temporal and spatial expression analysis of DEGs at different nodes and months. Blue indicates the top node, red indicates the center node and grey indicates the bottom node

Analyzing the FPKM values indicated a number of interesting trends in the expression of several DEGs at different nodes. In general, the expression of the same gene at different nodes might vary from Nov to Mar of the following year. However, there was a normalization phenomenon between Mar and Apr, i.e., independent of how it changed before, ultimately, it tended to be the same. This phenomenon was found in hormone metabolism, starch-sucrose metabolism, and photosynthesis pathway. For example, the expression line chart of *VvGA2OX6* (VIT_05s0077g00520) indicated that the time points at which the top, center, and bottom buds reached the FPKM pinnacle differed. Firstly, the top bud increased slowly to a small peak from Nov to Jan, the center bud decreased slightly from Nov to Dec, and then increased sharply to a small peak from Dec to Jan. At the same time, the bottom bud was at a stage of slow decline. Secondly, from Jan to Mar, the top and center buds decreased first and then showed a slight up-regulation. The bottom bud increased to a small peak and then decreased during this period, and the FPKM of all buds increased rapidly from Mar to Apr (Fig. 6, Supplementary Table 9). Moreover, the *VvXERICO* (VIT_12s0057g01330) expression of the top bud peaked in Jan, which was 1 month earlier than that of the center and bottom buds; however, after Feb, these three showed similar expression patterns (Fig. 6, Supplementary Table 9).

The expressions of starch-sucrose metabolism-associated genes, e.g., starch synthase 3 (*VvSS3*; VIT_10s0116g01730), *VvPHS2* (VIT_06s0004g06020) and AGPase (VIT_03s0038g04570), on the bottom bud and the center bud reached the first peak 1 month earlier than the top bud. Furthermore, the bottom bud then extended the down-regulation time, and the trend of the three remained roughly the same since Feb (Fig. 6, Supplementary Table 9).

The expression of the serine hydroxymethyl transferase (a photorespiration related enzyme) encoding gene (VIT_18s0001g04340) on the center buds peaked first in Dec, followed by the top buds, and finally the bottom buds, and then, the expression levels of all slightly decreased between Feb and Mar and then sharply declined (Fig. 6, Supplementary Table 9). Additionally, temporal and spatial differences of node expression could also be observed on *VvHVA22F* (VIT_17s0000g08730), *VvGH3.1* (VIT_03s0091g00310), and a fructokinase I encoding gene (VIT_01s0011g00240) (Fig. 6, Supplementary Table 9). Furthermore, another interesting phenomenon was found in the expression pattern of photosynthetic-related genes. The expression patterns of most genes were very similar in the bottom and the center buds, but there were always differences in time and expression levels between the bottom buds and the other two buds. Furthermore, the performance of this difference in different genes showed a certain degree of similarity, e.g., VIT_00s0207g00210, VIT_00s0125g00280, VIT_04s0044g01410, VIT_04s0023g00410, and VIT_06s0080g00920 (Fig. 6, Supplementary Table 9).

### The main regulatory pathways of three key dormant processes

**Fig. 7:**
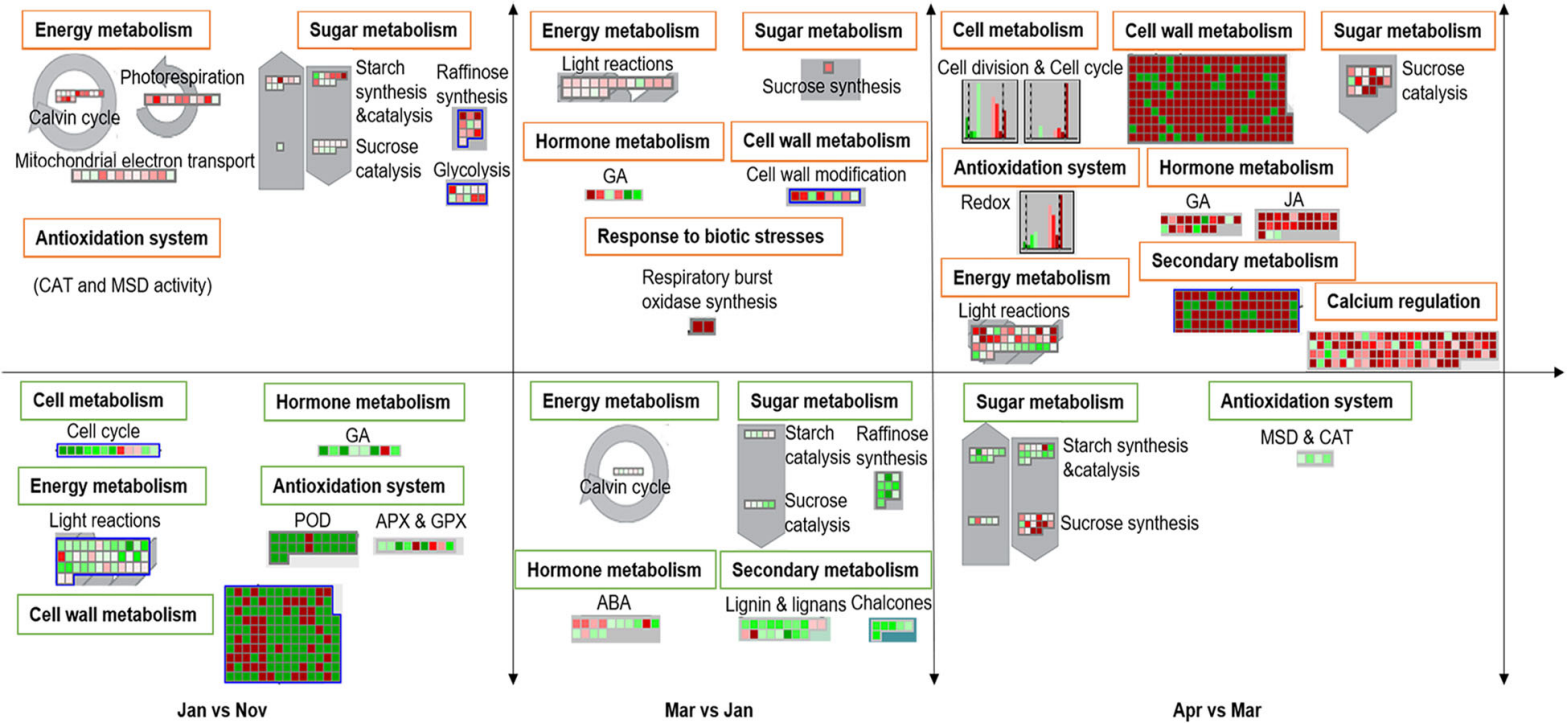
The main regulatory pathways of three key dormant processes. Red indicates up-regulation and green indicates down-regulation of each comparisons

Informed by these results, the 6 months of the dormancy period were divided into three main processes. Combined with Mapman analysis and pathways descriptions above, several main metabolic regulatory pathways were identified between one or 2 months. Between Nov and Jan, the main changes included up-regulation of energy metabolism (e.g., Calvin cycle, photorespiration, and mitochondrial electron transport), sugar metabolism (e.g., starch synthesis and catalysis, sucrose degradation, raffinose synthesis, and glycolysis), and antioxidation system (e.g., CAT and MSD activity). However, the main down-regulated pathways included cell cycle, cell wall metabolism, GA synthesis and signaling in hormone metabolism, energy metabolism (e.g., activity of PSI and PSII in light reactions), activity of antioxidant enzymes consisting of peroxidase (POD), glutathione peroxidase (GPX), and ascorbate peroxidase (APX) (Fig. 7). The comparative pair of Mar and Jan, included the main activated pathways of PSI and PSII activities, synthesis of sucrose in sugar metabolism, GA synthesis and signaling of hormone metabolism, cell wall modification, and response to biotic stresses (such as respiration burst oxidase synthesis). Down-regulated pathways included Calvin cycle of energy metabolism, sucrose and starch degradation and raffinose synthesis of sugar metabolism, ABA signaling, and secondary metabolism (e.g., synthesis of chalcones and lignin) (Fig. 7). When entering Apr, compared with Mar, most pathways associated with growth and development were reactivated, including cell cycle and division, cell wall synthesis and modification, sucrose degradation, activity of antioxidant enzymes except for CAT and MSD, GA and JA synthesis and signaling of hormone metabolism, photosynthesis of energy metabolism, various secondary metabolism (chalcones, phenylpropanoids, lignin, and simple phenols), and calcium regulation. Additionally, the main pathway repressed in Apr vs Mar was sugar metabolism (e.g., starch synthesis, starch catalysis, and sucrose synthesis) and the activities of CAT and MSD (Fig. 7).

## Discussion

### Critical dormancy transition time points

From the DEG numbers and their changing trends, identified via pair-wise comparisons, the minimum quantity was found in Feb vs Jan with similar expression changing trends, indicating a similar situation between Jan and Feb. Compared with Apr, the numbers of DEGs were very large in each month, followed by comparisons between Nov and each of the other months; however, the difference of Apr vs Nov was largest and smallest of all comparative combinations of their own, respectively. The maximum number was found in Apr vs Feb (10,032), followed by Apr vs Jan (9957), with only 75 genes difference. The number of DEGs in Apr vs Dec was 8137, and the difference was 1820 compared with that of Apr vs Jan, which was significantly larger than the previous pair. In addition, the numbers of DEGs in Mar vs Jan and Feb were 3191 and 1754, respectively, increased by nearly 45%. However, the statistical results indicated that the average minimum and maximum daily temperatures were all in January (1–7 °C), and the average daily length in January was 10.3 h, which was only 0.2 h longer than the shortest average day length in Dec. Starting from middle and late Mar, the average length exceeded 12 h. Previous studies showed that the blackcurrant has the least gene activity in the early dormancy stages and the maximum activity at budbreak [27]. According to the DEGs cluster result of the present study, high expression activity was found in Apr, and relatively low gene activity was found in Nov and Dec. Based on these phenomena, it can be speculated that Nov was the entering point of dormancy, Jan was the deepest dormancy period of buds, budbreak began in Mar, and part of the buds began to sprout in Apr. A similar observation period was also reported by Yamane [53], according to the percentage of bud-burst from October to Mar of the following year. In the Japanese apricot, bud dormancy began to release between late Dec and Jan; however, the bud-burst percentage increased continually, and the time to bud burst continued to shorten from Jan onwards; therefore, less dormancy was defined from Feb to Mar. The study of Liu et al. [10] also reported a similar model of division and comparison for dormancy stages, in which, the transcriptomes of ‘Suli’ pear buds collected on Nov 15, Dec 15, Jan 15, and Feb 15 were analyzed and compared.

### Changes in key metabolic pathways and expression of key genes

These sequencing results showed that DEGs are mainly enriched in redox balance, secondary metabolism, cellular function (e.g., cell wall metabolism, cell cycle, and cell division) and starch-sucrose metabolism. According to the climate status of the sampling period, the DEG numbers, and the result of cluster analysis, showed that Jan vs Nov (deepening dormancy), Mar vs Jan (non-deep dormancy period), and Apr vs Mar (dormancy releasing period) could be considered key periods of grape dormancy transition. Oxidative stress and its response play essential roles in bud dormancy release and bud-burst. It has been hypothesized that the reactivation of basic metabolic pathways and the subsequent generation of ROS are required to resume growth and cell division after dormancy [18]. H_2_O_2_ is a form of ROS, that has recently been reported to be associated with cell growth, cell division and other cellular processes [54–57] and ·OH may exert an active role in cell wall loosening [58]. It has been suggested that hydrogen cyanamide (HC) quickly decreases the activities of CAT and MSD, followed by increases of H_2_O_2_ and superoxide levels, which were concomitant with the onset of bud breaking in sweet cherry and grape [16, 59–62]. Similarly, the activities of CAT and MSD were up-regulated in Jan vs Nov, but were markedly repressed in Apr vs Mar, which was accompanied by the inhibition of *VvCAT2* and *VvMSD1* expression as dormancy began to release in Apr in the present study. However, in response to oxidative burst, expressions of *GPX*, *POD*, *GST*, *APX*, *Trxh*, and hypoxia related genes (such as sucrose synthase (*SuSy*)) were stimulated to scavenge ROS in dormancy-released buds [26, 36, 63]. This matches the findings of the present study, where the activities of these various antioxidant enzymes above were repressed in Jan vs Nov but were highly induced in Apr and DEGs also changed expression correspondingly. In addition, recent studies suggested that calcium signaling played an essential role in bud-burst with the reaction with ROS. NADPH oxidases regulate plant growth and development by producing ROS that control plant cell expansion via Ca^2+^ activation [64], and integrate calcium signaling and protein phosphorylation with increased ROS generation [65]. Several calcium signaling DEGs were also found to be highly expressed in Apr in this study, such as VIT_05s0020g04420, VIT_08s0040g00470, and VIT_01s0010g03020 (Supplementary Table 6).

The cessation of plant developmental processes such as cell cycle and cell division seems to be a hallmark of dormancy, while resumed vegetative growth is always accompanied by reactivated cell division in buds [42]. From Nov to Jan, with the onset and deepening of grape bud dormancy, cell cycle and cell division were suspended. However, in Apr vs Mar, these were extremely up-regulated, which was closely related to the activity of *CYCDs*. *CYCDs* were down-regulated as dormancy deepened, but were highly induced as buds began to sprout in Apr. A similar result has been observed in pear, where *CYCDs* expression was low during the transition phase from endodormancy to ecodormancy and then increased before sprouting [51]. These results indicated slower cell division and cell elongation in dormant buds compared with non-dormant buds, which further approved the recovery of bud cell activity after dormancy release.

Cell wall and secondary metabolism were also reported to play important roles in bud dormancy transition in grapes [20] and blackcurrant [27], and a close relationship between both was reported [20]. Greater phenylpropanoid abundance may be a result of resumed active growth in long-day buds, which may contribute to increased cell wall metabolism [20, 66]. Flavonoid compounds decreased in short day vs long day *Xanthium* [67] and enzymes relevant to the flavonoid biosynthesis showed more abundance in shoot tips of the long-day grape [66]. Secondary metabolism and corresponding DEGs such as *VvANS*, *VvF3H*, and *VvCHS*, as well as *VvCAD*, *Vv4CL*, and *VvCCoAMT*, were highly induced in Apr vs Mar but repressed in Mar vs Jan. While cell wall metabolism and corresponding DEGs (such as *Expansinm* and *VvXTH*) had highly induced expression in Apr vs Mar and Mar vs Jan, they were repressed during Nov and Jan. *Expansinm*, a gene involved in cell expansion due to its cell wall loosening activity, was up-regulated in the chilling-demand fulfillment period of the grapevine [25].

The role of carbohydrates in bud dormancy has also been reported in species such as grapes [20], peach [68], and kiwifruit [69]. Reserved carbohydrates are the main energy source for metabolisms that changed during the dormant period and budburst during spring [70]. During the dormancy progression, to resist the chilling winter conditions, part of the starch reserves are hydrolyzed into soluble sugars by starch degrading enzymes [71] and α-amylases have been reported to be up-regulated by cold stress in grapevine [72]. Similarly, starch catalysis and sucrose synthesis were up-regulated in Jan vs Nov and Mar vs Jan, respectively. Furthermore, β-amylase coding genes were highly expressed during Dec and Mar and peaked in Jan. Additionally, the sucrose catalysis process was markedly up-regulated coupled with high expression of sucrose-degrading genes in Apr vs Mar. This might be because sucrose was converted to hexoses (e.g., glucose and fructose), which provide carbon and energy for bud cells to synthetize various essential compounds for budbreak [70].

### Temporal and spatial expression differences of different node buds

Temporal and spatial differences of gene expression have been reported for *Brassica* [73] and *A. thaliana* [74]. However, the gene temporal and spatial expression patterns in different node buds of grape from endodormancy onset to release have not been reported to date. The annual branches of many grape varieties can differentiate into flower buds from the bottom node to the top node, and bloom and bear fruit the following year. Although significant differences were found in the development time of different node buds, significant differences were not found in the germination, inflorescence drawing, and flowering time [75]. This indicated that the differentiation process of grape branches differed from bottom to top, and finally, the phenomenon of ‘gradual synchronization’ appeared [75].

Several DEGs, with main functions of phytohormones synthesis and signaling (GA, ABA, and IAA), starch and sucrose metabolism, and photosynthesis exhibited noticeable temporal and spatial expression differences in different bud nodes, which might reach peaks or valleys at different times. For example, *GA2OX6* (VIT_05s0077g00520), *VvXERICO* (VIT_12s0057g01330), *GH3.1* (VIT_03s0091g00310), *VvSS3*(VIT_10s0116g01730), *VvPHS2* (VIT_06s0004g06020), and AGPase (VIT_03s0038g04570) had expression patterns on three nodes that likely differ from each other, or one of which might be far from the other two; however, independent of how they changed between Nov and Mar, they eventually showed a generally consistent expression trend between Mar and Apr, i.e., convergence.

Expression patterns of most different photosynthesis-related genes were found to be very similar in the bottom buds and the center buds. However, there were always differences in time and expression level between the bottom buds and the other two, and the performance of this changing pattern in different genes was surprisingly consistent.

Bud activity is coordinated at the whole-plant level but showed differences at the level of individual buds [76]. This might reflect a conspicuous difference of temporal and spatial expressions of several DEGs between different growth sites of grape buds during dormancy. The expression trends of the same genes in different node buds were basically identical, indicating that the same developmental process was conducted in different node buds; however, the gene expression level and time of different node buds were different [75]. Moreover, the changing trend of the expression level of different genes in different node buds during the dormancy period remained basically the same. This indicated that the development stage of each node bud was similar and entered a similar development process. Consequently, the dormancy period might be identified as the key stage of ‘gradual synchronization’ during the development of the buds at each node. Perhaps, such phenomena may occur in all organs or developmental stages throughout the life process of a plant. To explore these phenomena in more depth, a more intensive sampling mode could be adapted in the future, such as sampling once a week or once every 2 weeks to identify a more specific trend of change.

### Similarities and differences with a previous study

Coincidentally, the winter sampling times in the study of Díaz-Riquelme et al. [3] were also very similar to those used in the current study. The current study used finer sampling during winter, with additional sampling in Dec and Feb, mainly to observe the continuous changes of the metabolic pathways and the related gene expression levels, which happened within the buds from the initial stage of grape bud dormancy formation (early winter) to the beginning of dormancy release (early spring). In contrast, predecessors [3] also sampled during summer and early fall, drawing a picture of events, occurring during dormancy entrance and transition between endo- and eco-dormancy. This also provides ideas for future research related to dormancy in fruit trees. With regard to research technology, the current work used the RNA-seq technology while Díaz-Riquelme et al. [3] used microarray. RNA-seq showed more DEGs in general, providing more information for gene function analysis, while the biological mechanisms involved in the phases of dormancy were similar. Differences could also emerge because different genotypes were investigated by both studies. In addition, the current study provides novel insight about the temporal and spatial gene expression differences of different node buds along branches, sampled at the same time.

## Conclusions

This study provides novel insight into the genetic regulation of dormancy transition in the grapevine, and the adoption of next-generation sequencing (RNA-seq) technique shows key changes in gene expression during dormancy transition. The presented results enable the proposition of a bud dormancy regulating network model. Redox balance, secondary metabolites, cellular function (e.g., cell wall metabolism, cell cycle, and cell division), and carbohydrate metabolism, could interact with each other and respond to external factors to regulate the dormancy progress. Furthermore, a novel idea about ‘gradual synchronization’ in temporal and spatial expression of different node buds was also developed and will be further explored in the future.

## Methods

### Sample collection and weather recording

Three-year-old *Vitis vinifera* cv. ‘Rosario Bianco’ (‘Rosaki’ × ‘Muscat of Alexandria’) grapevines were cultured in the Jiangsu Agricultural Expo Garden in China (N32°0′41.99″, E119°15′7.11″) with permission from Jiangsu Vocational College of Agriculture and Forestry. After defoliation and dormancy entering of plants during November (Nov), 2016, 12 branches were randomly selected and cut and the same 30 to 40 branches on each plant were observed every time. To obtain clearer expression changing spectrums of grape bud dormancy and different bud nodes during dormancy and due to the ~ 30% germination rate in Apr, buds of different nodes were sampled every month from Nov to Apr of the next year. The buds were harvested from the bottom (B; the third, fourth, and fifth buds from the cordon), center (C; the eighth, ninth, and tenth buds from the cordon), and top (T; the fourteenth, fifteenth, and sixteenth buds from the cordon) of each branch in Nov and December (Dec) 20, 2016, January (Jan), February (Feb), March (Mar) 20 and Apr 8, 2017, respectively. At least 30 buds were collected from each node per month. The same node samples of the same sampling time were mixed, instantly frozen in liquid nitrogen and then stored at − 80 °C until RNA extraction. The temperature, humidity, and length of day were measured during each investigation and sampling, and the average day length and temperatures of the 5 days before and after the sampling day were calculated.

### Preparation for RNA-seq library

The Foregene RNA isolation kit (Foregene Co. Ltd., Chengdu, China) was used for total RNA extraction and RNA quality was checked using a 2100 Bioanalyzer (Agilent Technologies, Inc., Santa Clara, CA, USA). The total RNA of all samples collected from each dormancy stage was pooled into 36 samples (6 months × 3 growth positions × 2 replicates), which were sequenced on an Illumina HiSeq™ 2500 platform (Illumina, San Diego, CA, USA).

### Mapping of reads and analysis of differential gene expression

Clean reads were obtained by removing low-quality reads and reads containing adapter and more than 10% anonymous nucleotides (N) from raw sequence data (raw reads). The subsequently conducted analyses were all based on clean data with high quality. Clean reads were mapped to the *V. vinifera* reference genome (ftp://ftp.ensemblgenomes.org/pub/release-23/plants/gtf/vitis_vinifera/) by using the mapping software HISAT (version 2.0.4). The sequences obtained from Illumina sequencing were deposited in the NCBI Sequence Read Archive (accession number PRJNA488534).

HTSeq [77] (version 0.6.1) was utilized to count the reads mapped to each gene. According to the gene length and read count mapped to this gene, the expected number of Fragments Per Kilobase of transcript sequence per Millions base pairs sequenced (FPKM) of every gene was calculated. Differential expression analysis and calculations were conducted based on the count values of each transcript between libraries, using DESeq software (version 1.10.1) [78]. The thresholds for significant differences in transcript expression were the “adjusted p-value (padj) < 0.05” and the “|log_2_ fold-change (log_2_ FC) | ≥ 1”. Genes with FPKM < 0.3 were considered as not expressed and were therefore excluded in at least one group [79].

### Functional annotations of total DEGs during grape dormancy

When further comparing the differentially expressed genes (DEGs) between different months, two repeats were used in each of the three nodes per month, therefore, this was equivalent to six biological replicates per month. The expression patterns of DEGs at different bud nodes were equivalent to two repeats.

Gene ontology (GO) and Kyoto encyclopedia of genes and genomes (KEGG) pathway annotations were performed via Plant MetGenMAP tools [80] (http://bioinfo.bti.cornell.edu/cgi-bin/MetGenMAP/home.cgi) with thresholds of FDR ≤ 0.05 and *p*-value ≤0.05, respectively. Functional annotation of all DEGs was also performed by using MapMan (Vvnifera_145, http://mapman.gabipd.org/web/guest/mapmanstore) [81]. Heatmaps of gene expression levels in Nov, Dec, Jan, Feb, Mar, and Apr were obtained using Morpheus tools (https://software.broadinstitute.org/morpheus/).

### Prediction of novel genes and functional annotation

The genomic localization results of all sequencing reads data were assembled using Cufflinks software [82] (version 2.1.1), and Cuffcompare (a part of the Cufflinks package) was used to compare these results with the known *V. vinifera* reference genome to identify novel genes and their genomic localizations. The sequences of all novel genes were directly blasted via Blast2GO software [83] (version 2.7.1) to obtain their gene descriptions and functional annotations.

### Real-time quantitative PCR (RT-qPCR) validation of DEGs

Twenty genes were randomly selected to verify the expression patterns that were revealed by the RNA-seq technique using RT-qPCR. Based on the 3′ UTR sequence information, the gene specific RT-qPCR primer pairs were designed by Primer3 software (http://primer3.ut.ee/) [84]. With regard to the genes lacking 3′ UTR sequence information, primers were designed to anneal in the coding region. Purified RNA samples were reverse-transcribed using the Revert Aid™ First-Strand cDNA Synthesis Kit (Fermentas, Glen Burnie, MD, USA) following the manufacturer’s protocol. An Applied Biosystems® 7500 Real-Time PCR machine (Applied Biosystems, Foster City, CA, USA) was utilized for RT-qPCR. Each 20 μl reaction mix was composed of 10 μl EvaGreen 2× qPCR MasterMix-ROX (ABM, Richmond, BC, Canada), 2.0 μl cDNA sample, 0.6 μl forward primer, 0.6 μl reverse primer, and 6.8 μl nuclease-free H_2_O. The reaction program used the following procedures: 10 min (95 °C), followed by 35 cycles of 15 s at 95 °C and 1 min at 62 °C, with a final cooling to 4 °C. Each cDNA sample was used in triplicate for RT-qPCR analysis. The cycle thresholds (Ct) of the triplicate reactions for each tested gene were averaged, and then, these values were normalized to that of the *Actin* gene (AB073011, forward primer GGAAGCTGCGGGAATTCATGAG, reverse primer CCTTGATCTTCATGCTGCTGGG). The relative expression level of each gene was calculated via the 2^-ΔΔCT^ formula [85].

## Supplementary information

**Additional file 1: Figure S1.** Correlation between groups of comparisons. **Figure S2.** Mapman analysis of different pairs. (a) Metabolism overview of Mar vs Feb. (b) Metabolism overview of Feb vs Jan. (c) Metabolism overview of Jan vs Dec. (d) Metabolism overview of Dec vs Nov.

**Additional file 2: Table S1.** The information about RNA-Seq data. **Table S2.** Gene expression information. **Table S3.** FPKM values of all genes. **Table S4.** GO analytical data of each pair. **Table S5.** KEGG analytical data of each pair. **Table S6.** Mapman dataset. **Table S7.** Analysis of transcription factors. **Table S8.** Information about genomic localization and sequence length of all novel genes. **Table S9.** Analysis of temporal and spatial expression differences of genes at different nodes.

## Data Availability

The sequencing data are available in the NCBI Sequence Read Archive (SRA) database under the accession number PRJNA488534. All data generated or analyzed during this study are included in this published article (and its supplementary information files).

## Abbreviations

RNA-seq: RNA-sequencing
DEG: Differentially expressed gene
ABA: Abscisic acid
H_2_O_2_: Hydrogen peroxide
O_2_^−^: Superoxide anion radicals
OH: Hydroxyl radicals
iTRAQ: Isobaric tag for relative and absolute quantitation
TMT: Tandem mass tags
Nov: November
Dec: December
Jan: January
Feb: February
Mar: March
Apr: April
*V. vinifera*: *Vitis vinifera*
FPKM: Expected number of Fragments Per Kilobase of transcript sequence per Millions base pairs sequenced
padj: Adjusted *p*-value
log_2_ FC: log_2_ fold-change
Ct: Cycle threshold
GO: Gene ontology
KEGG: Kyoto encyclopedia of genes and genomes
RT-qPCR: Real-time quantitative PCR
BP: Biological process
CC: Cellular component
MF: Molecular function
ROS: Reactive oxygen species
GST: Glutathione-S-transferase
MT: Metallothionein
GA: Gibberellin
CTK: Cytokinin
ETH: Ethylene
JA: Jasmonate acid
SA: Salicylic acid
TF: Transcription factor
ABR1: AP2-like ABA repressor 1
PSI: Photosystem I
PSII: Photosystem II
LEA: Late embryogenesis abundant protein
HC: Hydrogen cyanamide
POD: Peroxidase
GPX: Glutathione peroxidase
APX: Ascorbate peroxidase
*A. thaliana*: *Arabidopsis thaliana*
IAA: indole-3-acetic acid
