# Supplementary figures and images for: Comparative transcriptome analysis provides insight into regulation pathways and temporal and spatial expression characteristics of grapevine (Vitis vinifera) dormant buds in different nodes
Source: BMC Plant Biol. 2020 Aug 26;20:390. doi: 10.1186/s12870-020-02583-1 (PMC7449092; doi:10.1186/s12870-020-02583-1)

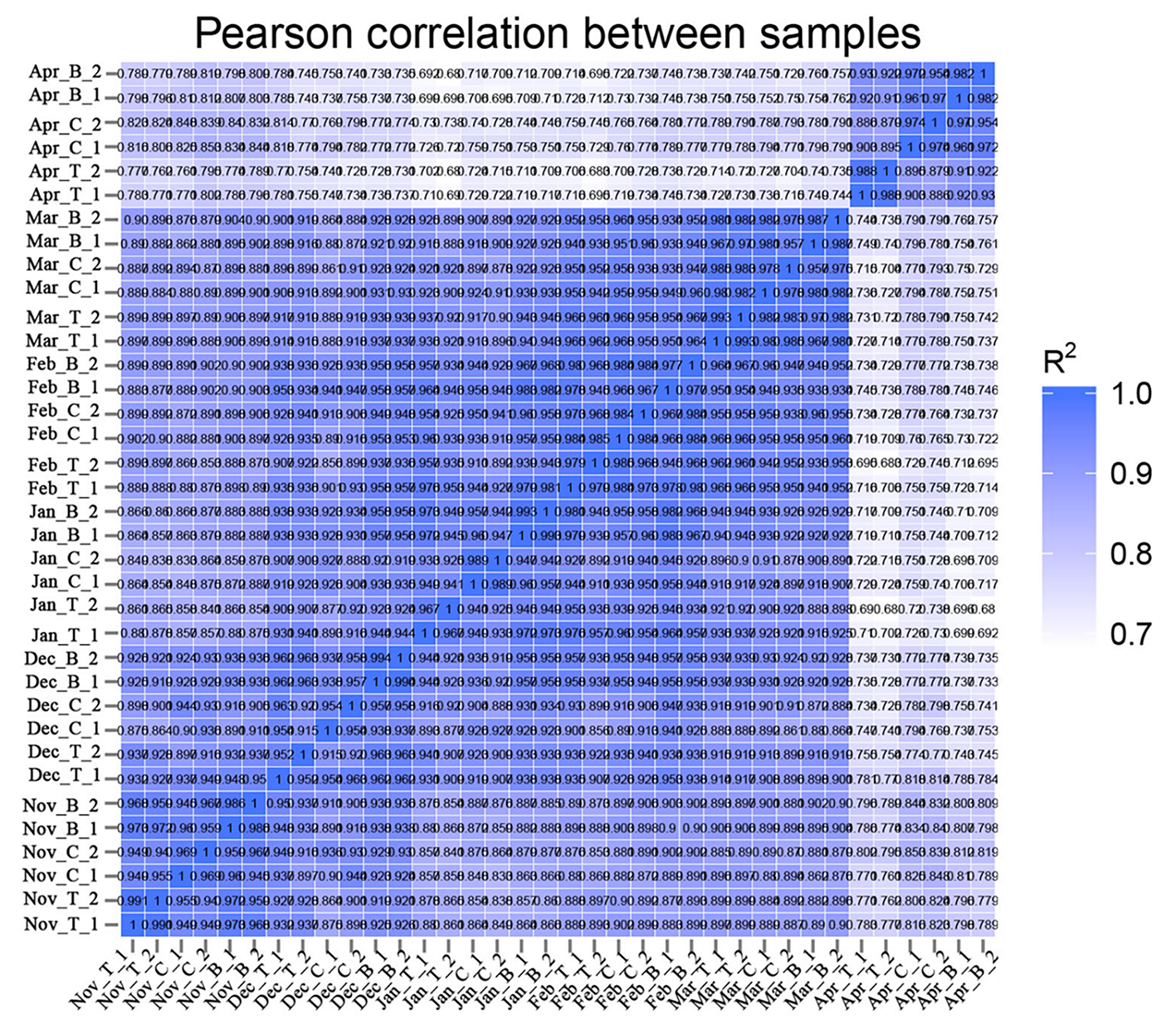

Supplement: Supplementary file 1 — Additional file 1: Figure S1. Correlation between groups of comparisons. Figure S2. Mapman analysis of different pairs. (a) Metabolism overview of Mar vs Feb. (b) Metabolism overview of Feb vs Jan. (c) Metabolism overview of Jan vs Dec. (d) Metabolism overview of Dec vs Nov. [file 12870_2020_2583_MOESM1_ESM.zip › Supplementary Figure 1_ESM.png]

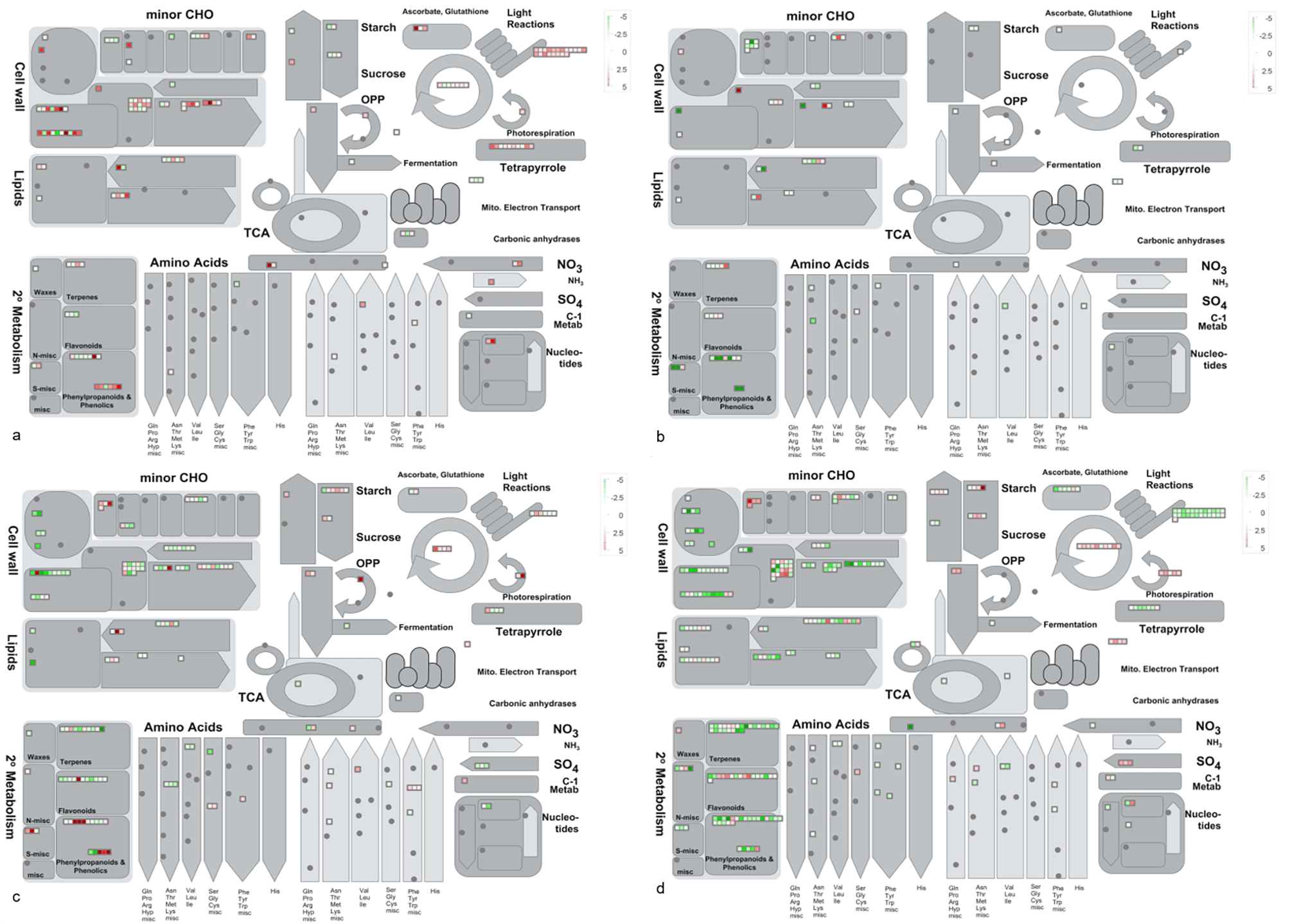

Supplement: Supplementary file 1 — Additional file 1: Figure S1. Correlation between groups of comparisons. Figure S2. Mapman analysis of different pairs. (a) Metabolism overview of Mar vs Feb. (b) Metabolism overview of Feb vs Jan. (c) Metabolism overview of Jan vs Dec. (d) Metabolism overview of Dec vs Nov. [file 12870_2020_2583_MOESM1_ESM.zip › Supplementary Figure 2_ESM.png]
